# Bis{benzyl 3-[(1*H*-indol-3-yl)methyl­idene]dithio­carbazato-κ^2^ *N* ^3^,*S*}palladium(II) pyridine disolvate

**DOI:** 10.1107/S1600536811001991

**Published:** 2011-01-22

**Authors:** Hamid Khaledi, Hapipah Mohd Ali

**Affiliations:** a Department of Chemistry, University of Malaya, 50603 Kuala Lumpur, Malaysia

## Abstract

The Pd^II^ ion in the title compound, [Pd(C_17_H_14_N_3_S_2_)_2_]·2C_5_H_5_N, is located on an inversion center and is four-coordinated by two of the deprotonated *N*,*S*-bidentate Schiff base ligands in a square-planar geometry. The dihedral angle between the aromatic ring planes within the ligand is 71.12 (9)°. The indole NH groups are bonded to the pyridine solvent mol­ecules *via* an N—H⋯N inter­action. The crystal structure is consolidated by inter­molecular C—H⋯S inter­actions.

## Related literature

For the analogous DMF disolvate Pd^II^ complex, see: Khaledi & Mohd Ali (2011 ▶). For a discussion of the coordination chemistry of indole-based *S*-benzyl­dithio­carbazones, see: Khaledi *et al.* (2011 ▶). 
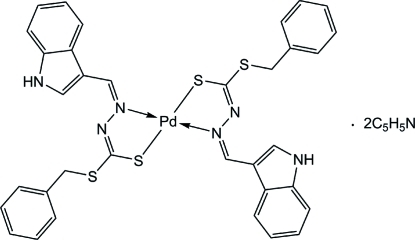


## Experimental

### 

#### Crystal data

[Pd(C_17_H_14_N_3_S_2_)_2_]·2C_5_H_5_N
*M* *_r_* = 913.46
Triclinic, 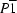

*a* = 9.9688 (2) Å
*b* = 10.5041 (2) Å
*c* = 10.9491 (2) Å
α = 62.534 (2)°
β = 78.494 (2)°
γ = 78.985 (2)°
*V* = 990.35 (3) Å^3^
*Z* = 1
Mo *K*α radiation
μ = 0.72 mm^−1^
*T* = 100 K
0.10 × 0.07 × 0.05 mm

#### Data collection

Bruker APEXII CCD diffractometer
Absorption correction: multi-scan (*SADABS*; Sheldrick, 1996 ▶) *T*_min_ = 0.931, *T*_max_ = 0.965
8128 measured reflections
3879 independent reflections
3045 reflections with *I* > 2σ(*I*)
*R*_int_ = 0.041

#### Refinement

*R*[*F* ^2^ > 2σ(*F* ^2^)] = 0.039
*wR*(*F* ^2^) = 0.070
*S* = 0.99
3879 reflections
262 parameters
1 restraint
H atoms treated by a mixture of independent and constrained refinement
Δρ_max_ = 0.73 e Å^−3^
Δρ_min_ = −1.03 e Å^−3^

### 

Data collection: *APEX2* (Bruker, 2007 ▶); cell refinement: *SAINT* (Bruker, 2007 ▶); data reduction: *SAINT*; program(s) used to solve structure: *SHELXS97* (Sheldrick, 2008 ▶); program(s) used to refine structure: *SHELXL97* (Sheldrick, 2008 ▶); molecular graphics: *X-SEED* (Barbour, 2001 ▶); software used to prepare material for publication: *SHELXL97* and *publCIF* (Westrip, 2010 ▶).

## Supplementary Material

Crystal structure: contains datablocks I, global. DOI: 10.1107/S1600536811001991/pv2380sup1.cif

Structure factors: contains datablocks I. DOI: 10.1107/S1600536811001991/pv2380Isup2.hkl

Additional supplementary materials:  crystallographic information; 3D view; checkCIF report

## Figures and Tables

**Table 1:** Hydrogen-bond geometry (Å, °)

*D*—H⋯*A*	*D*—H	H⋯*A*	*D*⋯*A*	*D*—H⋯*A*
N1—H1*N*⋯N4	0.86 (2)	1.96 (2)	2.808 (4)	171 (3)
C9—H9⋯S1^i^	0.95	2.58	3.267 (3)	130

Symmetry code: (i) 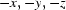.

**Fig. 1.**
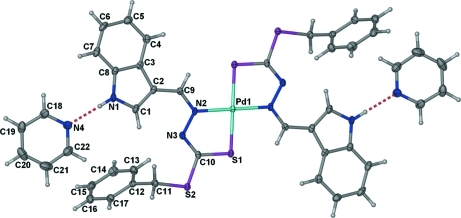
Thermal ellipsoid plot of the title compound at the 50% probability level. Hydrogen atoms are drawn as spheres of arbitrary radius. The unlabeled atoms are related to the labeled atoms by symmetry operation: -x, -y, -z.

## supplementary crystallographic information

### Comment

The crystal of the title compound was obtained from a pyridine solution of the Pd^II^ complex of indole-3-carbaldehyde *S*-benzyldithiocarbazone. Upon deprotonation, the Schiff base chelates the Pd^II^ ion in an *N,S*-bidentate bonding mode to form a five-membered ring with the metal center. The Pd^II^ ion, located on an inversion center, is four-coordinated by two of the Schiff base ligands in a square-planar geometry. The pyridine solvent molecules remain uncoordinated to the metal ion and are hydrogen bonded to indole NH groups. This is similar to what was observed in the structure of the analogous DMF solvate Pd^II^ complex (Khaledi & Mohd Ali, 2011). In contrast, the cadmium(II) complex of the Schiff base ligand in a pyridine solution gave an octahedral complex wherein two *trans*-pyridine molecules are coordinated to the metal center (Khaledi *et al.*, 2011). In the present structure, the aromatic ring planes within the ligand make a dihedral angle of 71.12 (9)°. The pyridine solvent ring is nearly coplanar with the indole ring, the dihedral angle between them being 11.39 (19)°. The structure is further consolidated by intramolecular interactions of the types C—H···S and C—H···N (Table 1).

### Experimental

The Schiff base ligand was prepared as reported previously (Khaledi *et al*., 2011). A solution of palladium(II) acetate (0.224 g, 1 mmol) in ethanol (30 ml) was added to an ethanolic solution (30 ml) of the ligand (0.65 g, 2 mmol) containing a few drops of triethylamine. The mixture was refluxed for an hour, then cooled to room temperature. The resulting brown solid was filtered, washed with cold ethanol and dried over siliga-gel. The crystals of the title compound were obtained by slow evaporation of a solution of the solid in pyridine.

### Refinement

The C-bound H atoms were placed at calculated positions (C–H 0.95–0.99 Å) and were treated as riding on their parent C atoms. The N-bound H atom was located in a difference Fourier map, and was refined with a distance restraint of N–H 0.88±0.02. For all H atoms, *U*_iso_(H) was set to 1.2 *U*_eq_(carrier atom).

### Crystal data

**Table:**

[Pd(C_17_H_14_N_3_S_2_)_2_]·2C_5_H_5_N	*Z* = 1
*M**_r_* = 913.46	*F*(000) = 468
Triclinic, *P*1	*D*_x_ = 1.532 Mg m^−^^3^
Hall symbol: -P 1	Mo *K*α radiation, λ = 0.71073 Å
*a* = 9.9688 (2) Å	Cell parameters from 2496 reflections
*b* = 10.5041 (2) Å	θ = 2.2–27.4°
*c* = 10.9491 (2) Å	µ = 0.72 mm^−^^1^
α = 62.534 (2)°	*T* = 100 K
β = 78.494 (2)°	Block, red
γ = 78.985 (2)°	0.10 × 0.07 × 0.05 mm
*V* = 990.35 (3) Å^3^	

### Data collection

**Table:**

Bruker APEXII CCD diffractometer	3879 independent reflections
Radiation source: fine-focus sealed tube	3045 reflections with *I* > 2σ(*I*)
graphite	*R*_int_ = 0.041
φ and ω scans	θ_max_ = 26.0°, θ_min_ = 2.1°
Absorption correction: multi-scan (*SADABS*; Sheldrick, 1996)	*h* = −11→12
*T*_min_ = 0.931, *T*_max_ = 0.965	*k* = −12→12
8128 measured reflections	*l* = −13→13

### Refinement

**Table:**

Refinement on *F*^2^	Primary atom site location: structure-invariant direct methods
Least-squares matrix: full	Secondary atom site location: difference Fourier map
*R*[*F*^2^ > 2σ(*F*^2^)] = 0.039	Hydrogen site location: inferred from neighbouring sites
*wR*(*F*^2^) = 0.070	H atoms treated by a mixture of independent and constrained refinement
*S* = 0.99	*w* = 1/[σ^2^(*F*_o_^2^) + (0.0263*P*)^2^] where *P* = (*F*_o_^2^ + 2*F*_c_^2^)/3
3879 reflections	(Δ/σ)_max_ = 0.001
262 parameters	Δρ_max_ = 0.73 e Å^−^^3^
1 restraint	Δρ_min_ = −1.03 e Å^−^^3^

### Special details

**Table:**

Geometry. All e.s.d.'s (except the e.s.d. in the dihedral angle between two l.s. planes) are estimated using the full covariance matrix. The cell e.s.d.'s are taken into account individually in the estimation of e.s.d.'s in distances, angles and torsion angles; correlations between e.s.d.'s in cell parameters are only used when they are defined by crystal symmetry. An approximate (isotropic) treatment of cell e.s.d.'s is used for estimating e.s.d.'s involving l.s. planes.
Refinement. Refinement of *F*^2^ against ALL reflections. The weighted *R*-factor *wR* and goodness of fit *S* are based on *F*^2^, conventional *R*-factors *R* are based on *F*, with *F* set to zero for negative *F*^2^. The threshold expression of *F*^2^ > σ(*F*^2^) is used only for calculating *R*-factors(gt) *etc*. and is not relevant to the choice of reflections for refinement. *R*-factors based on *F*^2^ are statistically about twice as large as those based on *F*, and *R*- factors based on ALL data will be even larger.

### Fractional atomic coordinates and isotropic or equivalent isotropic displacement parameters (Å^2^)

**Table:**

	*x*	*y*	*z*	*U*_iso_*/*U*_eq_	
Pd1	0.0000	0.0000	0.0000	0.01415 (11)	
S1	0.22662 (8)	−0.07299 (8)	0.03590 (8)	0.01946 (19)	
S2	0.39814 (8)	0.01851 (8)	0.15858 (8)	0.01993 (19)	
N1	−0.0326 (3)	0.4905 (3)	0.2098 (3)	0.0209 (6)	
H1N	0.014 (3)	0.528 (3)	0.240 (3)	0.025*	
N2	0.0161 (3)	0.1372 (2)	0.0784 (2)	0.0161 (6)	
N3	0.1382 (3)	0.1317 (2)	0.1281 (2)	0.0168 (6)	
C1	0.0172 (3)	0.3767 (3)	0.1816 (3)	0.0211 (7)	
H1	0.1081	0.3286	0.1913	0.025*	
C2	−0.0838 (3)	0.3404 (3)	0.1364 (3)	0.0175 (7)	
C3	−0.2052 (3)	0.4404 (3)	0.1403 (3)	0.0175 (7)	
C4	−0.3413 (3)	0.4581 (3)	0.1129 (3)	0.0207 (7)	
H4	−0.3696	0.3974	0.0822	0.025*	
C5	−0.4324 (3)	0.5660 (3)	0.1317 (3)	0.0247 (8)	
H5	−0.5249	0.5785	0.1146	0.030*	
C6	−0.3925 (4)	0.6577 (3)	0.1754 (3)	0.0257 (8)	
H6	−0.4580	0.7315	0.1863	0.031*	
C7	−0.2600 (3)	0.6428 (3)	0.2026 (3)	0.0236 (8)	
H7	−0.2324	0.7052	0.2317	0.028*	
C8	−0.1682 (3)	0.5327 (3)	0.1860 (3)	0.0185 (7)	
C9	−0.0809 (3)	0.2353 (3)	0.0870 (3)	0.0161 (7)	
H9	−0.1638	0.2379	0.0551	0.019*	
C10	0.2357 (3)	0.0394 (3)	0.1092 (3)	0.0155 (7)	
C11	0.3916 (3)	0.1657 (3)	0.2031 (3)	0.0192 (7)	
H11A	0.4837	0.2004	0.1736	0.023*	
H11B	0.3258	0.2461	0.1484	0.023*	
C12	0.3507 (3)	0.1328 (3)	0.3543 (3)	0.0180 (7)	
C13	0.2418 (3)	0.0539 (3)	0.4334 (3)	0.0243 (7)	
H13	0.1898	0.0208	0.3920	0.029*	
C14	0.2083 (4)	0.0229 (3)	0.5724 (3)	0.0283 (8)	
H14	0.1344	−0.0324	0.6260	0.034*	
C15	0.2820 (4)	0.0720 (3)	0.6337 (3)	0.0289 (8)	
H15	0.2590	0.0507	0.7291	0.035*	
C16	0.3883 (4)	0.1516 (3)	0.5552 (3)	0.0265 (8)	
H16	0.4383	0.1868	0.5964	0.032*	
C17	0.4238 (3)	0.1814 (3)	0.4174 (3)	0.0209 (7)	
H17	0.4988	0.2356	0.3650	0.025*	
N4	0.1071 (3)	0.5947 (3)	0.3379 (3)	0.0247 (6)	
C18	0.0482 (4)	0.6915 (3)	0.3856 (3)	0.0293 (8)	
H18	−0.0442	0.7315	0.3704	0.035*	
C19	0.1138 (4)	0.7360 (4)	0.4550 (3)	0.0322 (8)	
H19	0.0671	0.8044	0.4875	0.039*	
C20	0.2470 (4)	0.6811 (4)	0.4771 (4)	0.0392 (10)	
H20	0.2951	0.7107	0.5245	0.047*	
C21	0.3098 (4)	0.5814 (4)	0.4286 (4)	0.0435 (10)	
H21	0.4026	0.5412	0.4416	0.052*	
C22	0.2362 (4)	0.5409 (4)	0.3610 (3)	0.0327 (9)	
H22	0.2799	0.4710	0.3293	0.039*	

### Atomic displacement parameters (Å^2^)

**Table:**

	*U*^11^	*U*^22^	*U*^33^	*U*^12^	*U*^13^	*U*^23^
Pd1	0.0175 (2)	0.01085 (18)	0.0166 (2)	−0.00293 (15)	−0.00176 (15)	−0.00774 (15)
S1	0.0206 (5)	0.0190 (4)	0.0251 (4)	−0.0007 (4)	−0.0042 (4)	−0.0151 (4)
S2	0.0200 (5)	0.0204 (4)	0.0249 (4)	0.0001 (4)	−0.0052 (4)	−0.0147 (4)
N1	0.0240 (17)	0.0187 (14)	0.0272 (15)	−0.0053 (12)	−0.0004 (12)	−0.0160 (12)
N2	0.0220 (15)	0.0108 (12)	0.0192 (13)	−0.0021 (11)	−0.0027 (11)	−0.0094 (10)
N3	0.0201 (15)	0.0144 (13)	0.0184 (13)	−0.0034 (12)	−0.0033 (11)	−0.0084 (11)
C1	0.0260 (19)	0.0155 (15)	0.0219 (17)	−0.0009 (14)	−0.0011 (14)	−0.0096 (13)
C2	0.0228 (18)	0.0120 (14)	0.0159 (15)	−0.0032 (13)	0.0021 (13)	−0.0060 (12)
C3	0.0207 (18)	0.0128 (15)	0.0157 (16)	−0.0033 (14)	0.0030 (13)	−0.0050 (12)
C4	0.026 (2)	0.0179 (16)	0.0185 (16)	−0.0060 (14)	−0.0002 (14)	−0.0081 (13)
C5	0.0251 (19)	0.0237 (17)	0.0209 (17)	0.0025 (15)	−0.0028 (14)	−0.0082 (14)
C6	0.032 (2)	0.0178 (16)	0.0204 (17)	0.0060 (15)	0.0017 (15)	−0.0080 (13)
C7	0.034 (2)	0.0141 (15)	0.0213 (17)	0.0000 (15)	0.0000 (15)	−0.0093 (13)
C8	0.0207 (18)	0.0165 (15)	0.0173 (16)	−0.0043 (14)	0.0028 (13)	−0.0079 (13)
C9	0.0170 (17)	0.0153 (15)	0.0149 (15)	−0.0052 (13)	−0.0009 (13)	−0.0051 (12)
C10	0.0210 (18)	0.0133 (14)	0.0115 (15)	−0.0045 (13)	−0.0006 (13)	−0.0046 (12)
C11	0.0186 (18)	0.0183 (15)	0.0240 (17)	−0.0048 (14)	−0.0022 (14)	−0.0111 (13)
C12	0.0211 (18)	0.0139 (15)	0.0189 (16)	0.0027 (13)	−0.0056 (13)	−0.0077 (13)
C13	0.0253 (19)	0.0274 (17)	0.0255 (18)	−0.0069 (15)	−0.0023 (15)	−0.0149 (15)
C14	0.030 (2)	0.0265 (17)	0.0221 (17)	−0.0056 (16)	0.0026 (14)	−0.0066 (14)
C15	0.039 (2)	0.0242 (17)	0.0201 (18)	0.0099 (17)	−0.0075 (16)	−0.0106 (15)
C16	0.035 (2)	0.0217 (17)	0.0277 (18)	0.0053 (16)	−0.0130 (16)	−0.0149 (15)
C17	0.0237 (19)	0.0162 (15)	0.0246 (17)	−0.0011 (14)	−0.0053 (14)	−0.0102 (13)
N4	0.0237 (16)	0.0202 (14)	0.0313 (16)	−0.0052 (13)	−0.0042 (13)	−0.0110 (12)
C18	0.025 (2)	0.0247 (18)	0.041 (2)	−0.0061 (16)	0.0012 (16)	−0.0175 (16)
C19	0.038 (2)	0.032 (2)	0.033 (2)	−0.0160 (18)	0.0082 (17)	−0.0209 (16)
C20	0.047 (3)	0.051 (2)	0.025 (2)	−0.027 (2)	−0.0019 (18)	−0.0140 (18)
C21	0.030 (2)	0.057 (3)	0.034 (2)	0.001 (2)	−0.0108 (18)	−0.011 (2)
C22	0.034 (2)	0.0276 (19)	0.031 (2)	0.0034 (17)	−0.0009 (17)	−0.0120 (16)

### Geometric parameters (Å, °)

**Table:**

Pd1—N2^i^	2.031 (2)	C9—H9	0.9500
Pd1—N2	2.031 (2)	C11—C12	1.510 (4)
Pd1—S1^i^	2.2936 (8)	C11—H11A	0.9900
Pd1—S1	2.2936 (8)	C11—H11B	0.9900
S1—C10	1.729 (3)	C12—C13	1.387 (4)
S2—C10	1.751 (3)	C12—C17	1.393 (4)
S2—C11	1.810 (3)	C13—C14	1.384 (4)
N1—C1	1.350 (4)	C13—H13	0.9500
N1—C8	1.377 (4)	C14—C15	1.384 (5)
N1—H1N	0.857 (18)	C14—H14	0.9500
N2—C9	1.296 (3)	C15—C16	1.369 (5)
N2—N3	1.411 (3)	C15—H15	0.9500
N3—C10	1.294 (3)	C16—C17	1.376 (4)
C1—C2	1.387 (4)	C16—H16	0.9500
C1—H1	0.9500	C17—H17	0.9500
C2—C9	1.430 (4)	N4—C22	1.326 (4)
C2—C3	1.450 (4)	N4—C18	1.339 (4)
C3—C8	1.408 (4)	C18—C19	1.365 (5)
C3—C4	1.408 (4)	C18—H18	0.9500
C4—C5	1.378 (4)	C19—C20	1.365 (5)
C4—H4	0.9500	C19—H19	0.9500
C5—C6	1.400 (4)	C20—C21	1.378 (5)
C5—H5	0.9500	C20—H20	0.9500
C6—C7	1.376 (5)	C21—C22	1.376 (5)
C6—H6	0.9500	C21—H21	0.9500
C7—C8	1.390 (4)	C22—H22	0.9500
C7—H7	0.9500		
			
N2^i^—Pd1—N2	180.0	N3—C10—S2	120.5 (2)
N2^i^—Pd1—S1^i^	83.22 (7)	S1—C10—S2	112.65 (16)
N2—Pd1—S1^i^	96.78 (7)	C12—C11—S2	116.6 (2)
N2^i^—Pd1—S1	96.78 (7)	C12—C11—H11A	108.1
N2—Pd1—S1	83.22 (7)	S2—C11—H11A	108.1
S1^i^—Pd1—S1	180.0	C12—C11—H11B	108.1
C10—S1—Pd1	95.95 (11)	S2—C11—H11B	108.1
C10—S2—C11	104.39 (14)	H11A—C11—H11B	107.3
C1—N1—C8	109.9 (3)	C13—C12—C17	118.5 (3)
C1—N1—H1N	124 (2)	C13—C12—C11	121.7 (3)
C8—N1—H1N	126 (2)	C17—C12—C11	119.8 (3)
C9—N2—N3	114.6 (2)	C14—C13—C12	120.4 (3)
C9—N2—Pd1	124.4 (2)	C14—C13—H13	119.8
N3—N2—Pd1	121.06 (17)	C12—C13—H13	119.8
C10—N3—N2	112.8 (2)	C13—C14—C15	120.5 (3)
N1—C1—C2	110.1 (3)	C13—C14—H14	119.8
N1—C1—H1	125.0	C15—C14—H14	119.8
C2—C1—H1	125.0	C16—C15—C14	119.2 (3)
C1—C2—C9	131.8 (3)	C16—C15—H15	120.4
C1—C2—C3	105.7 (3)	C14—C15—H15	120.4
C9—C2—C3	122.4 (3)	C15—C16—C17	120.9 (3)
C8—C3—C4	118.9 (3)	C15—C16—H16	119.5
C8—C3—C2	106.7 (3)	C17—C16—H16	119.5
C4—C3—C2	134.3 (3)	C16—C17—C12	120.5 (3)
C5—C4—C3	118.1 (3)	C16—C17—H17	119.7
C5—C4—H4	121.0	C12—C17—H17	119.7
C3—C4—H4	121.0	C22—N4—C18	116.7 (3)
C4—C5—C6	121.9 (3)	N4—C18—C19	123.6 (3)
C4—C5—H5	119.1	N4—C18—H18	118.2
C6—C5—H5	119.1	C19—C18—H18	118.2
C7—C6—C5	121.1 (3)	C18—C19—C20	119.3 (3)
C7—C6—H6	119.4	C18—C19—H19	120.4
C5—C6—H6	119.4	C20—C19—H19	120.4
C6—C7—C8	117.3 (3)	C19—C20—C21	118.1 (4)
C6—C7—H7	121.4	C19—C20—H20	120.9
C8—C7—H7	121.4	C21—C20—H20	120.9
N1—C8—C7	129.7 (3)	C22—C21—C20	119.1 (4)
N1—C8—C3	107.6 (2)	C22—C21—H21	120.5
C7—C8—C3	122.7 (3)	C20—C21—H21	120.5
N2—C9—C2	130.7 (3)	N4—C22—C21	123.2 (3)
N2—C9—H9	114.7	N4—C22—H22	118.4
C2—C9—H9	114.7	C21—C22—H22	118.4
N3—C10—S1	126.8 (2)		

Symmetry codes: (i) −*x*, −*y*, −*z*.

### Hydrogen-bond geometry (Å, °)

**Table:**

*D*—H···*A*	*D*—H	H···*A*	*D*···*A*	*D*—H···*A*
N1—H1N···N4	0.86 (2)	1.96 (2)	2.808 (4)	171 (3)
C9—H9···S1^i^	0.95	2.58	3.267 (3)	130
C1—H1···N3	0.95	2.42	2.889 (4)	110
C11—H11B···N3	0.99	2.50	2.937 (4)	106

Symmetry codes: (i) −*x*, −*y*, −*z*.
